# Ossification of Transverse Ligament of Atlas Causing Cervical Myelopathy: A Case Report and Review of the Literature

**DOI:** 10.1155/2011/238748

**Published:** 2011-09-19

**Authors:** Tatsuro Sasaji, Chikashi Kawahara, Fujio Matsumoto

**Affiliations:** Department of Orthopaedic Surgery, Tohoku Rosai Hospital, 4-3-21 Dainohara, Aoba-ku, Sendai 981-8563, Japan

## Abstract

A case of ossification of transverse ligament of atlas (TLA) is reported. A 76-year-old female suffered from a transverse type myelopathy was successfully treated by posterior decompression. Dynamic lateral plain radiographs showed irreducible atlantoaxial subluxation (AAS). A computed tomogram revealed ossified mass compatible to ossification of TLA. Coalition of the atlantooccipital joints and osteoarthritis of the atlantoaxial joints with degenerated dens was also revealed. Magnetic resonance imaging showed compressed spinal cord at C1 level by the ossification of TLA and AAS. We suggest a mechanism of ossification of TLA as follows: hypertrophied dens and stress to the atlantoaxial joints caused by coalition of atlantooccipital joints could make forward shift of atlas leading to irreducible AAS, and continuous tension given to TLA from irreducible AAS would result in hypertrophied and ossification of TLA.

## 1. Introduction

A pseudotumor behind dens is known to be a factor associated with cervical myelopathy at the atlantoaxial region. The pathogenesis of pseudotumor is thought to be a granulation around transverse ligament of atlas (TLA). There have been only four reports about ossification of TLA so far, and the present condition could be a rare cause of cervical myelopathy at upper cervical spine [1–4]. We experienced a case of ossification of TLA. We report details of radiological findings and inference of mechanism of the ossification of TLA from current and previously reported cases.

## 2. Case Report

A 76-year-old woman had been suffered from numbness and clumsiness in both upper limbs and gait disturbance for six months before presentation.

### 2.1. Neurological Examination

She was diagnosed as a transverse type cervical myelopathy without neck pain. Her neck motion was severely restricted especially in rotation. All of the laboratory findings were within normal limits.

### 2.2. Radiological Findings

**Figure 1:**
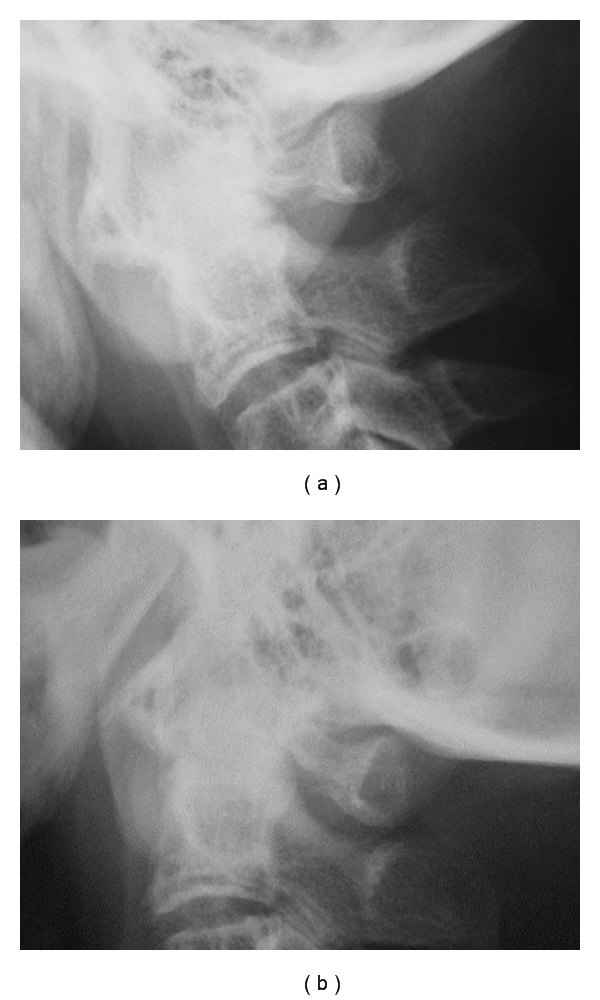
Preoperative dynamic lateral plain radiographs. Flexion view (a) and extension (b) view. Preoperative plain radiographs in flexed position (a) and in extended position (b) showed ossified mass behind dens and irreducible atlantoaxial subluxation. The atlantodental interval is 6 mm and the space available for the spinal cord is 7 mm in each positions.

**Figure 2:**
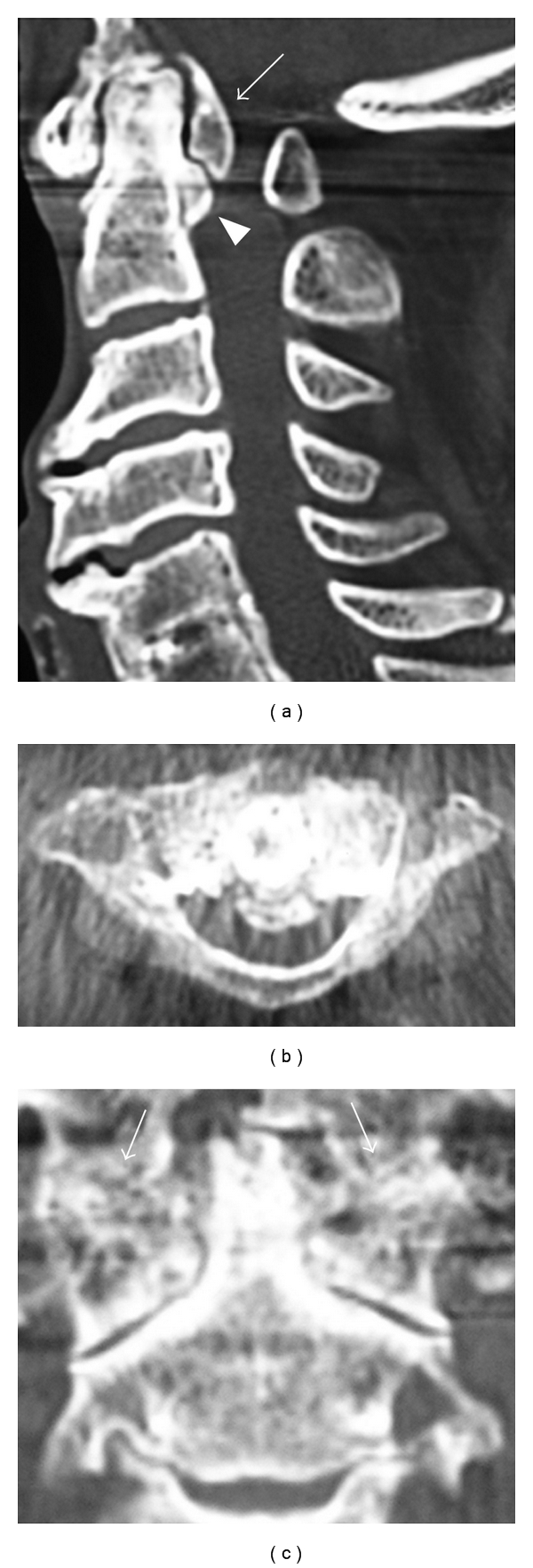
Reconstructed computed tomogram (CT). (a) Sagittal reconstructed CT. Ossified mass behind dens (arrow) and osteophyte continuing from the dens (arrowhead) can be seen. The dens itself is degenerated and hypertrophied. (b) Axial CT. Most of the spinal canal is occupied by ossification of transverse ligament of atlas. (c) Coronal reconstructed CT. The joint space of atlantoaxial joint and the spaces between dens and lateral masses of atlas were narrowed. Coalition of bilateral atlantooccipital joints can be seen (arrow).

**Figure 3:**
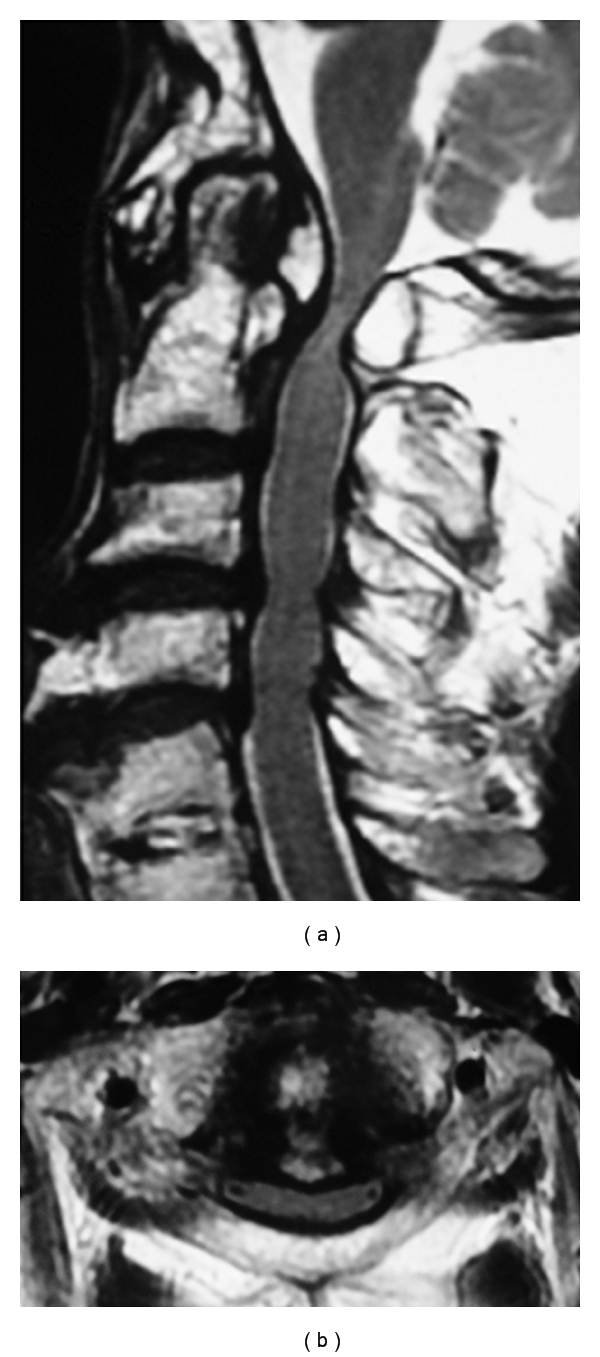
Preoperative MRI on T2-weighted image. (a) Sagittal plane. The spinal cord is compressed at the level of C1, C3-4, and C4-5, with high intensities within the spinal cord at C1 level. (b) Axial plane. The spinal cord is compressed by the mass behind dens and posterior arch of the atlas.

Dynamic lateral plain radiographs of cervical spine revealed irreducible atlantoaxial subluxation (AAS) (Figure 1). A sagittal reconstructed computed tomogram (CT) revealed the ossified mass behind dens and osteophyte continuing to dens. Dens and anterior arch of atlas were sclerotic, and dens itself was hypertrophied. There was no ossification of posterior longitudinal ligament (Figure 2(a)). CT also revealed ossified mass compatible to ossification of TLA. A space between dens and posterior arch decreased (Figure 2(b)). Dens and lateral masses of atlas were sclerotic and space around dens decreased in a coronal reconstructed CT. Coalition of bilateral atlantooccipital joints was seen (Figure 2(c)). A sagittal planes of magnetic resonance imaging (MRI) showed compressed spinal cord with high intensities within the spinal cord by the mass behind dens. The mass showed high intensities on T2-weighted images. The spinal cord was compressed between the mass and posterior arch of atlas, and there was no subarachnoid space around the spinal cord. The spinal cord was also compressed at the level of C3-4 and C4-5 (Figure 3(a)). Axial planes of MRI also showed compressed spinal cord at the level of C1 between the mass and posterior arch of atlas (Figure 3(b)).

### 2.3. Operation

Posterior arch of the atlas, C3, and C4 lamina were explored through a posterior approach. Resection of the posterior arch of atlas and laminectomy of C3 and C4 were performed using burr. After the decompression, the dura mater became pulsant and the decompression was confirmed by an ultrasonography.

### 2.4. Postoperative Course

**Figure 4:**
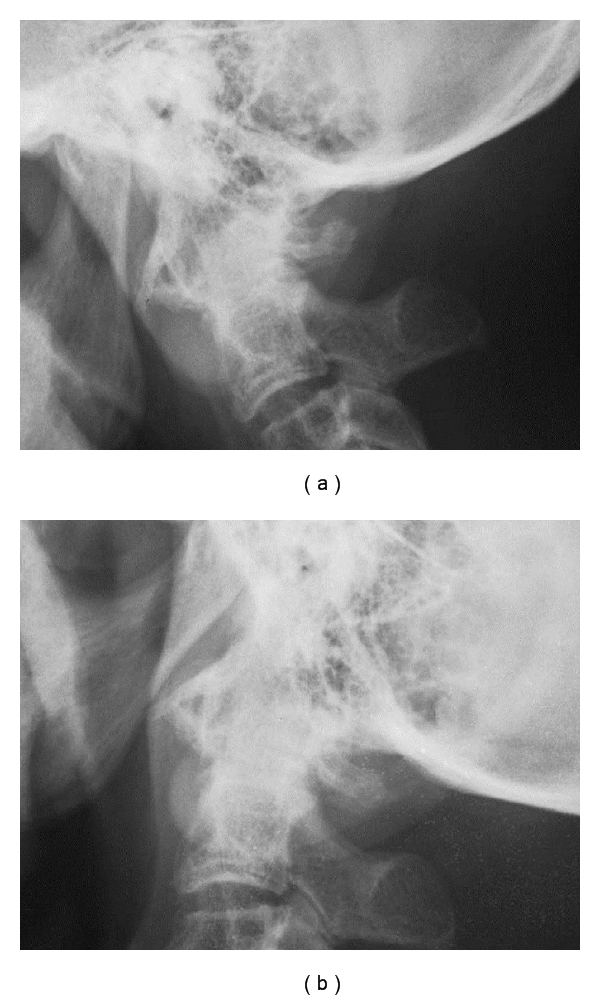
Postoperative dynamic lateral plain radiographs. Flexion (a) and extension (b) view. Atlantoaxial subluxation had not progressed.

**Figure 5:**
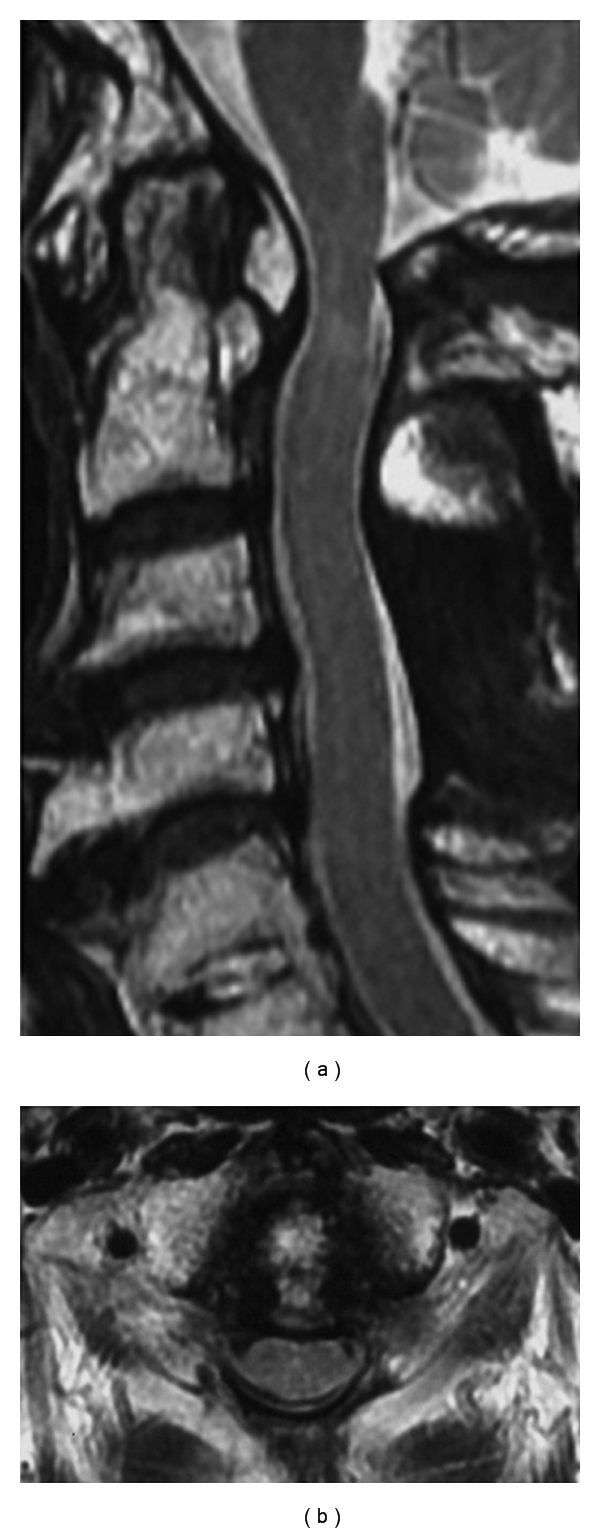
Postoperative MRI on T2-weighted image. Sagittal (a) and axial (b) plane. The spinal cord had been decompressed.

Her numbness and clumsiness of both upper limbs and gait disturbance were improved soon after the operation. At eighteen months after surgery, she returned to a normal daily life without neck pain. Follow-up plain lateral radiographs showed no deterioration of AAS after surgery (Figures 4(a) and 4(b)). The follow-up MRI revealed an adequate decompressed spinal cord (Figures 5(a) and 5(b)).

## 3. Discussion

There have been only four reports concerning ossification of TLA [1–4]. Characteristic features of patients with ossification of TLA were elderly population, AAS, and hypertrophied dens. AAS were recognized in three cases, and hypertrophied dens were recognized in two cases. Hayashi and colleagues described that a trauma of 12 years ago led to ossification of TLA, but they did not describe a mechanism of this ossification in detail [1]. The mechanism of this ossification is still unclear from the previous literatures.

 Irreducible AAS and osteoarthritis at atlantoaxial joints with hypertrophied dens were seen in the current case similar to the previously reported cases. A coalition of atlantooccipital joints was also recognized only in the current case. Remnants of the atlantooccipital joints were seen in a coronal reconstructed CT, and so we diagnosed not as an assimilation of atlas but as a coalition caused by degeneration. According to Detlef and Walter, an assimilation of the atlas caused AAS [5]. Greenberg, Hagiwara, and colleagues suggested a mechanism that atlantoaxial joint compensated the function of stabilized atlantooccipital joint and a greater stress on TLA results in stretching and leading to AAS [6, 7]. Coalition of atlantooccipital joints is different from an assimilation of atlas, but it may cause AAS as the adjacent segment degeneration by similar mechanism. On the basis of these findings, we suggested mechanism of ossification of TLA as follows: hypertrophied dens and coalition of atlantooccipital joints make forward shift of the atlas leading to irreducible AAS. Continuous stress given to TLA by irreducible AAS would result in hypertrophied and ossification of TLA leading to spinal cord compression.

 The posterior decompression was performed for current case, because there was no instability at occipito-atlantoaxial complex. We adopted the same procedure with previous reports and had no instability and complaint postoperatively.
